# Dysbiosis and inflammation in periodontitis: synergism and implications for treatment

**DOI:** 10.1080/20002297.2017.1325198

**Published:** 2017-05-25

**Authors:** George Hajishengallis

**Affiliations:** ^a^ University of Pennsylvania, Philadelphia, PA, USA

## Abstract

Recent advances from microbiome and host response mechanistic studies indicate that periodontitis is not a bacterial infection in the classical sense but rather results from dysbiosis owing to a breakdown in host-microbe homeostasis. Accordingly, periodontitis is induced in susceptible hosts by a polymicrobial community, in which different members have distinct roles that converge synergistically to enhance colonization, nutrient procurement and persistence in an inflammatory environment. Keystone pathogens – the colonization of which is facilitated by accessory pathogens – initially subvert the host response leading to the emergence of a dysbiotic microbiota, in which pathobionts overactivate the inflammatory response and cause periodontal tissue destruction. Inflammation and dysbiosis positively reinforce each other because inflammatory tissue breakdown products are used as nutrients by the dysbiotic microbiota, thereby contributing to its persistence and chronic inflammation. These concepts have important implications for novel therapeutic approaches, including strategies for microbial community manipulation and targeted modulation of the host response to limit destructive inflammation and reverse the microbial immune subversive tactics that fuel dysbiosis.

